# NOXA/MCL-1 axis determines cell-death decision between apoptosis and a GSDME-dependent cell death associated with production of inflammatory cytokines upon treatment of breast cancer cells with antimitotics

**DOI:** 10.1038/s41419-026-08965-x

**Published:** 2026-06-11

**Authors:** Alison Dumont, Fabien Gautier, Vanessa Josso, Quentin Batard, Aurélie Moreau, Mario Campone, Philippe Juin, Sophie Barillé-Nion

**Affiliations:** 1 CRCI2NA, Inserm 1307, CNRS UMR 6075, Nantes Université, Université Angers, Equipe Labellisée LIGUE Contre le Cancer, Nantes, France; 2 Nantes Université, INSERM, Center for Research in Transplantation and Translational Immunology, UMR 1064, ITUN, F-44000 Nantes, France; 3 ICO, René Gauducheau, Saint-Herblain, 44 France

**Keywords:** Breast cancer, Cell death

## Abstract

Understanding how malignant cells respond to chemotherapy is essential to prevent the development of resistance and to improve the efficiency of anticancer drugs. Antimitotic treatment in breast cancer cells increases NOXA, an endogenous inhibitor of the survival protein MCL-1. We herein show that whereas the extent of cell death is determined by the compensatory survival BCL-xL, NOXA/MCL-1 balance determines the modality of cell death demise. Indeed, genetic inactivation of NOXA, which reinforces the pro-survival function of MCL-1 in cancer cells, only postpones death upon exposure to Taxol and BCL-xL antagonism with A1331852. It directs the morphology of dying cells towards a phenotype characterized by cell swelling and balloon-like bubbling, regulated by Caspase-3 and GSDME, indicating that NOXA and MCL-1 might function as negative and positive, respectively, regulators of this cell death mode. Importantly, a comparative analysis of secretomes from NOXA-proficient and NOXA-deficient cancer cells treated with Taxol revealed variations in the production of inflammatory cytokines, including IL-1α, IL-1β, and IL-18. Thus, induction of apoptosis or a vacuole-associated and inflammatory cell death, in breast cancer cells by antimitotic treatment, relies on pore-forming protein GSDME expression but also on a fine balance within the BCL2 family network.

## Introduction

The initial objective of chemotherapy is to directly eradicate tumor cells, operating under the assumption that inducing their demise would result in the complete elimination of tumors and the recovery of patients. Recent studies on tumor cell death have unveiled two critical additional insights. First, individual cell death is not merely an endpoint when it comes to treat tumor cell populations in link with microenvironmental cells, as it carries valuable information for the tumor microenvironment. Second, there exists a diversity of interconnected pathways leading to cell death so that the same drug may trigger different modes of cell death in distinct cellular backgrounds. Depending on the specific cell death process undergone, dying cells release various signals into their microenvironment [1, 2]. These signals, constitutive or inducible, in turn, lead to differential tumor responses [3, 4], as they may trigger potent co-stimulatory signals, that either support or inhibit intrinsic tumor response to anticancer therapy or antitumoral response. Consequently, the modalities of therapy-induced cell death may be harnessed to provoke an effective antitumoral immune response, involving T-cell immunity, referred to as immunogenic cell death [1, 5].

Cytotoxic chemotherapies traditionally target rapidly dividing tumor cells and induce acute stress in affected cells. In response, these cells trigger damage repair pathways or, when overwhelmed, trigger self-destruct mechanisms. The outcome depends significantly on the intensity of acute stress and can lead to either cell survival or programmed cell death [4]. The cellular response varies according to the type of stress and the cellular context, involving distinct molecular pathways and the release of information [6, 7]. Beside well-characterized apoptotic pathways [8, 9], recent studies have identified and decoded pyroptosis as a cell death process initially engaged in immune cells during infection and relying on gasdermin (GSDM) family of proteins [10], mainly membrane pore-forming effectors GSDMD and GSDME, associated with the release of inflammatory cellular contents involved in regulation of essential innate immune processes and cancer cell response to chemotherapies [11, 12].

Accumulating evidence points to cell death as a potent regulator of inflammation, even during cancer therapies. Initially, apoptotic cells were considered immunologically silent due to their rapid clearance by phagocytes. In contrast, pyroptotic or necroptotic lytic cell death processes trigger an inflammatory response [3, 13]. Notably, mitochondria play a central role in determining whether a cell lives or dies, by modulating metabolic and respiratory activities as well as orchestrating regulated cell death (reviewed in [14]). The integrity of the outer mitochondrial membrane is governed by the BCL2 family proteins (including survival proteins such as MCL-1 and BCL-xL) that regulate its permeabilization (MOMP) [7, 8] and by the release of cytochrome-c (cyto-c) through activated BAX and BAK pores, which then activate the caspase-9-dependent apoptosome, to execute MOMP leading to the execution of the intrinsic apoptotic pathway. Additionally, when apoptotic caspase activities are inhibited, MOMP triggers pro-inflammatory signaling through the release of mitochondrial DNA, activating the cGAS/STING pathway, ultimately leading to the activation of the type I-IFN and NF-kB pathways [15, 16]. Recent studies have also highlighted that antimitotic therapies activate the cGAS/STING pathway, in response to chemo-induced micronuclei [17], in particular in breast cancer cells where a cascade serves as a catalyst for a proapoptotic secretome within tumors, enhancing the cytotoxic effects of the drug [18]. Of interest, this signaling activation enhances the response to genotoxic treatments and immunotherapy [19]. However, when chronic, the intrinsic cGAS/STING activation relying on chromosome instability (CIN) may also promote metastasis as reported by Bakhoum and colleagues, at least in immune-compromised mice [20]. This effect was in fact suppressed in immune competent mice, revealing noncell autonomous effects of STING activation upon CIN [21].

Understanding how tumor cells respond to chemotherapy-induced stress and the information they release into their microenvironment upon survival or upon activation of a certain mode of cell death, is crucial for unraveling the complex interplay that affects tumor progression or elimination. In this study, we explored the biological effects of antimitotic therapy in breast cancers, with the aim of better characterizing the stress and information released during cell death triggered by this treatment. We recently showed that antimitotics induce NOXA expression in breast cancer cell populations, by both cell autonomous and noncell autonomous mechanisms [18]. Induced NOXA, as an endogenous inhibitor of MCL-1, enhances apoptotic load on the compensatory BCL-xL so that cell death is enhanced upon co-treatment with a selective antagonist of BCL-xL that allows the production of autocrine signals by stressed cells. We herein reveal that, in fact, NOXA favors rapid apoptotic cell death at the expense of a delayed vacuole-associated cell death that relies on GSDME. Our results reveal that antimitotic treatment induces the release of pro-inflammatory IL1-related cytokines, specifically IL-1α, IL-1β, and IL-18, in breast cancer cell lines and human primary breast tumors. Importantly, the release of these cytokines depends on the specific cell death pathway engaged during antimitotic treatment and favors NK cells' proliferation. Thus, manipulation of NOXA or GSDME functions holds the potential to promote an inflammation process upon chemotherapy treatment, and thus to potentially redirect the immune response towards an antitumor response and antitumoral activity.

## Materials and methods

### Cells lines and reagents

MDA-MB-231 and MDA-MB-468 cell lines were purchased from ATCC and cultured in Dulbecco’s Modified Eagle Medium (DMEM) supplemented with 2 mM glutamine, 1% penicillin/streptomycin and 5% fetal bovine serum (FBS) (Eurobio, Courtaboeuf, France). MCL-1 and GSDME overexpressing cell lines were established by viral infection with retroviruses containing vector (plvx) and corresponding cDNA. For the CRISPR-Cas9-induced knock-out (KO) or knock-down (KD) cell lines, sgRNA sequences targeting human genes were designed using the CRISPR design tool (http://crispor.tefor.net). The guide sequences described in Table 1 were cloned into the plentiCRISPRV2 vector from Feng Zhang (Addgene plasmid #52961). All cells were selected using 1 μg/ml puromycin, and KO or KD were confirmed by immunoblot analysis.

**Table 1:** Guide sequences used in CRISPR-Cas9-based generation of KO cell lines.

Gene	Sequence (5’ → 3’)
Human *PMAIP1(NOXA)*	AGTAGAAAAGGGCGACAACC
Human *BAX*	AGTAGAAAAGGGCGACAACC
Human *BAK*	GCCATGCTGGTAGACGTGTA
Human *DFNA5(GSDME)*	GTCGGACTTTGTGAAATACG
Human *GSDMD*	ACAGGCAAAGATCGCAGGCG
Human *CASP1*	GCTCCCTAGAAGAAGCTCAA
Human *CASP3*	ATGTCGATGCAGCAAACCTC

For the preparation of conditioned media (CM), 3 × 10^6^ cells were treated as indicated during 24 h in 100 mm plates, washed three times with PBS and cultured in DMEM without FBS for 48 additional hours. CM were then collected, centrifuged (2000 rpm,10 min) and concentrated using a polyethersulfone membrane to retain proteins above 3 kDa.

In vitro treatments were used at the following concentrations: 100 nM A1331852 (ApexBio, Houston, TX, USA), 500 nM S63845 (Selleckchem, Houston, TX, USA), 70 nM Taxol (Sigma-Aldrich, St Quentin Fallavier, France), 5 μM Q-VD-OPh (R&D Systems, Abingdon, UK).

### Kinetic quantification of cell death

Kinetic cell death analysis was determined using an Incucyte Zoom imaging system (Sartorius, Göttingen, Germany). Cells were plated in 1 mM thymidin-containing medium overnight for cell-cycle synchronization. Cells were then treated with Taxol (70 nM) or/and A1331852 (100 nM) in the presence of S63845 (500 nM) or Q-VD-OPh (5μM) or not, and propidium Iodide (PI) 10 μg/mL (Miltenyi Biotec, Germany) was then added. Plates were imaged every 60 or 120 min during 48 h. The quantitative analysis was done using Incucyte cell-by-cell analysis software module (Sartorius). All experiments were repeated at least three times, and one representative is shown. The qualitative analysis of cell death mode was evaluated on at least 30 dying cells in each condition, based on dye staining and/or on the formation of blebs and/or a unique vacuole bubbling during the cell death process.

### Cell death and cyto-c release assays by flow cytometry

Cell death analysis was evaluated by staining cells with Annexin V-FITC and PI, according to the manufacturer’s instructions. Cyto-c release was evaluated using the cyto-c antibody (560263, BD Biosciences) after fixation/permeabilization. Flow cytometry analysis was performed on FACS Accuri C6plus (BD Biosciences, Le Pont de Claix, France).

### Immunoblot analysis

Cellular proteins were obtained from cell lysates with RIPA buffer (89901, Thermo Fisher Scientific, Villebon Courtaboeuf, France) or from concentrated CM, before separation on SDS-PAGE and transfer to nitrocellulose membranes. Membranes were then incubated with primary antibodies (Table 2) 1 h at room temperature. Then, the membranes were incubated with the appropriate secondary antibodies for 1 h at room temperature. Immobilon Forte Western HRP substrate kit (WBLUF0100, Millipore, Marne-la-Coquette, France) was used for immunoblot revelation on the Fusion-FX 7 system (Vilber, Marne-la-Vallée, France). Original uncropped immunoblots are provided in the Supplementary Materials.

**Table 2:** Primary antibodies used for immunoblot analysis.

Protein	Reference	Supplier	Working dilution
Actin	MAB1501	EMD Millipore	1/2000
BAK	3814S	Cell Signaling Technology	1/1000
BAX	5023S	Cell Signaling Technology	1/1000
Caspase-3	9662S	Cell Signaling Technology	1/1000
GSDMD	HPA044487	Atlas Antibody	1/1000
GSDME	ab215191	Abcam	1/1000
IL-18	ab207323	Abcam	1/500
MCL-1	94296S	Cell Signaling Technology	1/1000
NOXA	ab13654	Abcam	1/500
ERK1/2	9102	Cell Signaling Technology	1/1000
p-ERK1/2	4370	Cell Signaling Technology	1/1000

### Caspase3/7 activity measurement

Caspase3/7 activity was quantified in cell lysates using the Kit Caspase-Glo® 3/7 Assay Systems (G8091, Promega, France). Luminescence measurement was performed using the lumino/fluorometer Mithras LB940 (Berthold Technologies, France).

### Quantification of secreted cytokines

Multiplex analysis of IL-1α, IL-1β, IL-6, IL-8 and CXCL1 secretion by MDA-MB-231 cell, was determined in cell supernatants using LegendPlex assays according to the manufacturer’s protocol (reference 437015 and personalized LegendPlex panel, Biolegend, Amsterdam, The Netherlands). Flow cytometry analysis was performed on an Aurora Cytek Spectral cytometer (Cytek, Amsterdam, The Netherlands). Levels of IL1β in cell supernatants were determined by ELISA assay according to the manufacturer’s protocol (437015, Biolegend). Cytokine concentrations were normalized to total protein in corresponding cell protein lysates.

### Transcriptomic analysis

For RT-qPCR experiments, total RNA was isolated using Nucleospin RNA plus (740990.50, Macherey Nagel, Hoerdt, France) and transcribed into cDNA by Maxima First Strand cDNA synthesis Kit (K1642, Thermo Scientific, Illkirch, France). Quantitative RT-PCR (qPCR) was performed using the EurobioGreen qPCR Mix Lo-Rox (GAEMMXOS1L-8T, Eurobio, Les Ulis, France) with qTOWER (Analytik-jena, Jena, Germany). Reaction was done in 10 μl final with 4 ng RNA equivalent of cDNA and 150 nM primers described in Table 3).

**Table 3:** Primers used in RT-qPCR analysis.

Gene	Primers
*IL1A*	TGTGACTGCCCAAGATGAAG/CTTAGTGCCGTGAGTTTCCC
*IL1B*	TGGCAATGAGGATGACTTGT/GGAAAGAAGGTGCTCAGGTC
*IL18*	ATTACTTTGGCAAGCTTGAATCTAAATTATCAGT/TACCTCTAGGCTGGCTATCTTTATACATACT
*ACTB*	AGAAAATCTGGCACCACACC/CAGAGGCGTACAGGGATAGC

For Nanostring analysis of breast tumors, fresh human breast cancer samples were sliced and incubated for 48 h with Taxol (700 nM) before mRNA extraction, as described in [18]. Gene expression was quantified with the NanoString nCounter platform in the nCounter human (255 genes) inflammatory panel (NanoString Technologies) using 50 ng of total RNA from patient-derived luminal tumor explants after a 48 h ex vivo incubation with Taxol 700 nM or not (as described in ref. [18]). The code set was hybridized with the RNA overnight at 65 °C. RNA transcripts were immobilized and counted using the NanoString nCounter Sprint. RCC files were read using the Nanostring R package. Positive control normalization and housekeeping genes normalization were done according to Gene Expression Data Analysis Guideline instructions (https://nanostring.com/wp-content/uploads/Gene_Expression_Data_Analysis_Guidelines.pdf). Normalized data were then processed using standard R workflows.

### Statistical analysis

All experiments were conducted at least three times in independent experiments, and results are presented as mean values ± SEM of biological replicates. The two ways-ANOVA test and the Student’s *t*-test were used for statistical analysis with GraphPad Prism 10.5.0 Software. Error bars represent standard deviation (SEM). The symbols correspond to a *P* value inferior to *0.05, **0.01, ***0.001, and ****0,0001

## Results

### NOXA favors apoptosis at the expense of a vacuole-associated cell death mode in triple-negative breast cancer cells in response to cytotoxic antimitotic chemotherapy

**Fig. 1:**
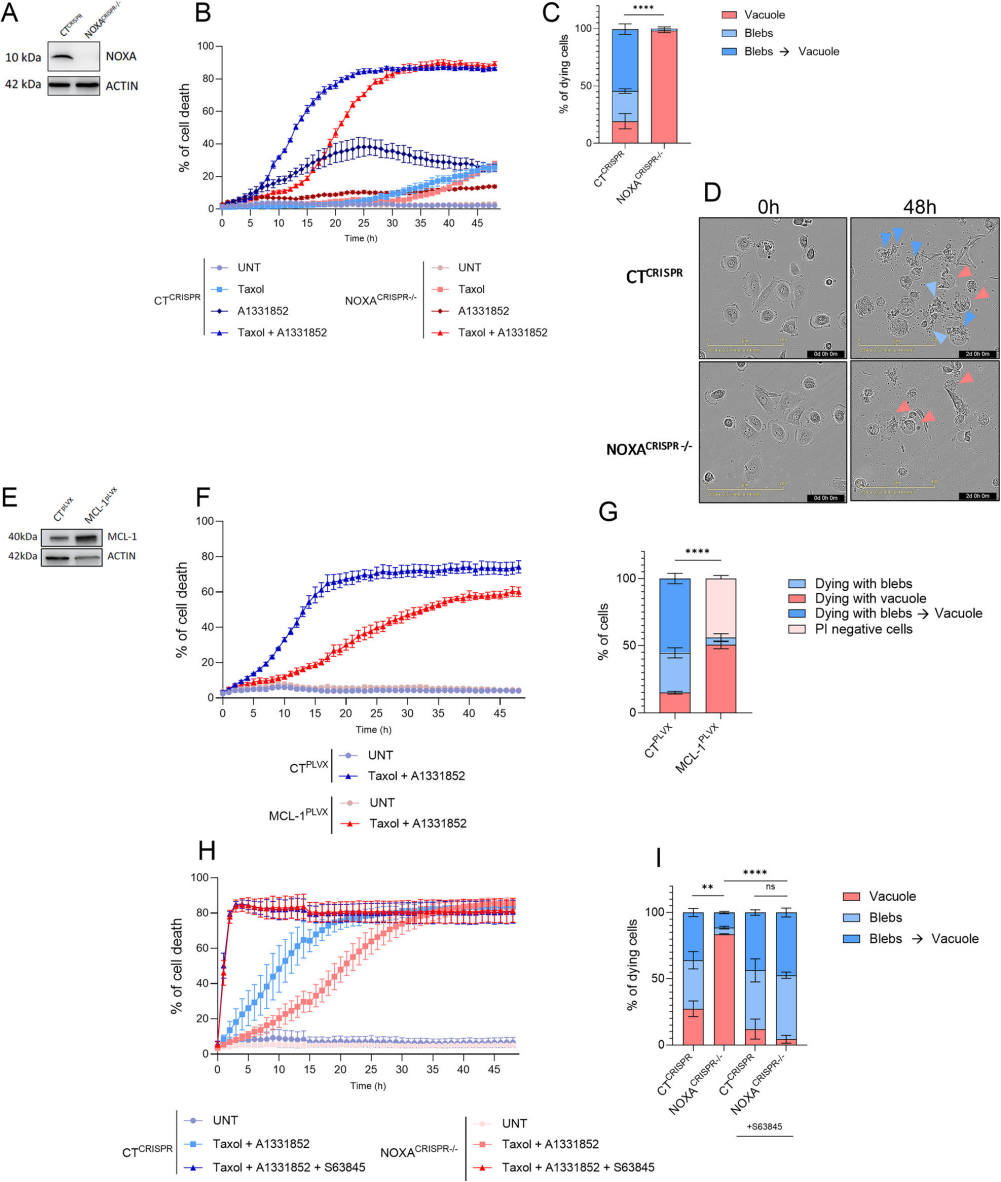
The NOXA/MCL-1 axis determines the mode of cell death induced by Taxol treatment combined with BCL-xL inhibition. **A**, **E** Immunoblot analysis of NOXA expression in CT^CRISPR^ NOXA^CRISPR−/−^(**A**) and MCL-1 expression in CT^PLVX^ and MCL-1^PLVX^ (**E**) MDA-MB-231 cells. **B**, **F**, **H** Kinetics of single cell death representative analysis in untreated (UNT) or treated by Taxol (70 nm) or the BCL-xL inhibitor A1331852 (100 nm) alone or combined **B**, **F** plus MCL-1 inhibitor S63845 (500 nM) **H** during 48 h, in CT^CRISPR^ and NOXA^CRISPR−/−^ (**B**, **H**) or in CT^PLVX^ and MCL-1^PLVX^ (**F**) MDA-MB-231 cells. **C**, **G**, **I** % of cells dying with blebs either associated with a secondary vacuole or not, or with an initial unique vacuole without blebs, and % of PI negative surviving cells, after a 48-h exposure to Taxol + A1331852 treatment in CT^CRISPR^ and NOXA^CRISPR−/−^ (**C**, **I**) or in CT^PLVX^ and MCL-1^PLVX^ (**G**) MDA-MB-231 cells. **D** Illustrative images at 0 and 48 h of combined treatment with blue arrows pointing blebs-associated cell death and red arrows pointing prominently vacuole-associated cell death without blebs, in CT^CRISPR^ (upper) and NOXA^CRISPR−/−^ (lower) MDA-MB-231 cells. All experiments were conducted at least three times in independent experiments, and results are presented as representative ones using mean values ± SEM.

To explore the extent to which NOXA contributes to cell death mode and its mode of execution in response to antimitotic agents, we used videomicroscopy to track each individual cell response to treatments. We first analyzed single cell death morphologies and kinetic changes arising in triple-negative breast cancer (TNBC) cell line MDA-MB-231 functionally deleted of NOXA by inactivating PMAIP1 coding gene by CRISPR-Cas9 (NOXA^CRISPR−/−^) or not (CT^CRISPR^), in response to the antimitotic Taxol combined to the BCL-xL specific BH3-mimetic A1331852, using propidium Iodide as cell viability dye. NOXA gene inactivation (Fig. 1A) not only led to delayed cell death occurrence when cancer cells were exposed to the cytotoxic treatment, as reported in ref. [18] (Fig. 1B). Strikingly, NOXA inactivation also completely shifted cell death mode towards a process characterized by the appearance of a ballooning morphology inherent to the initial formation of a unique cytoplasmic vacuole, in most of the cells (95%) (Fig. 1C, D), potentially evocating a pyroptotic-like process as described in refs. [22, 23] or secondary necrosis as reported in ref. [24], that we call hereafter a vacuole-associated cell death. In contrast, control cells (CT^CRISPR^ cells), that produced a majority (80%) of the initial apoptotic-like process (characterized by blebbing initiation and formation of apoptotic corpses) evolving or not towards an eventual ballooning process in the residual main corps, as illustrated in Fig. 1D.

MCL-1 overexpression (Fig. 1E) efficiently protected cells from death since analysis of single cell fates indicated that an average of 45% of the cells were still alive at the end of the experiments, compared to none in control cells. At the same time, MCL-1 promoted the vacuole-associated cell death engagement in the 55% dying cells (Fig. 1F, G), as did NOXA depletion. Conversely, antagonizing MCL-1 with its specific BH3-mimetic S63845, restored the ability of NOXA-depleted cells to undergo fast apoptosis at the expense of vacuole-associated cell death, as did the control cells (Fig. 1H, I). These results argue that MCL-1 and NOXA, as an endogenous MCL-1 antagonist, functionally mimicked by S63845, favor a slow vacuole-associated cell death mode or a rapid apoptotic cell death process, respectively.

### Preferential vacuole-associated cell death fate relies on MOMP and CASP3 activity

**Fig. 2:**
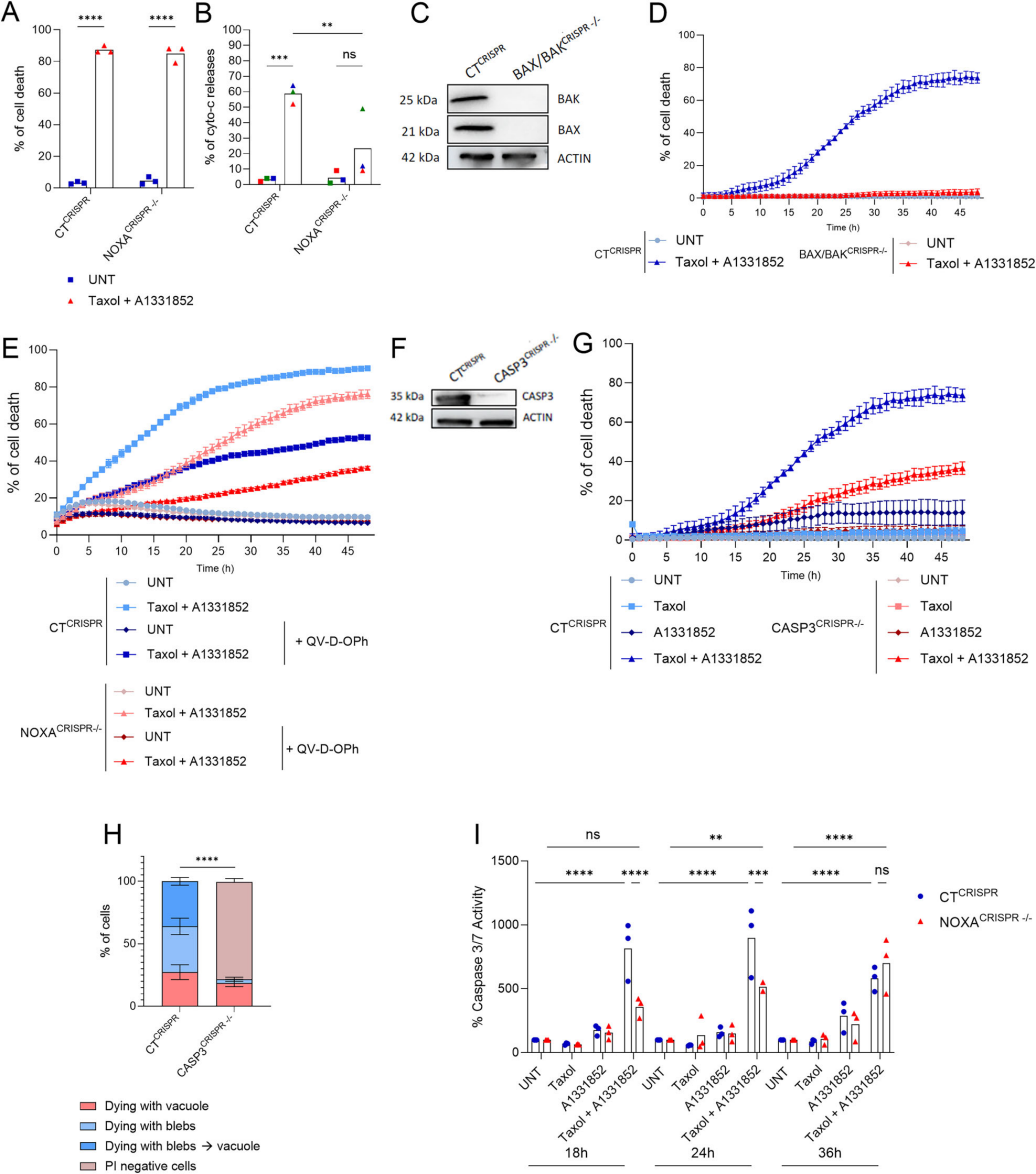
MOMP and casp3 activation are required for both apoptotic and pyroptotic-like cell death. **A** Analysis of cell death by flow cytometry in untreated (UNT) or treated with Taxol in combination with A1331852 during 36 h, in CT^CRISPR^ and NOXA^CRISPR−/^^−^ MDA-MB-231 cells. **B** Analysis of cyto-c release by flow cytometry in the same cells and conditions. **C** Immunoblot analysis of BAX and BAK expression in CT^CRISPR^ and double KO BAX/BAK^CRISPR−/−^ MDA-MB-231 cells. **D** Kinetics of single cell death representative analysis, in untreated (UNT) or treated by Taxol (70 nm) and BCL-xL inhibitor A1331852 (100 nm) during 48 h, in CT^CRISPR^ and double KO BAX/BAK^CRISPR−/−^ MDA-MB-231. **E** Kinetic quantification of cell death during treatment by Taxol and A1331852 combination during 48 h, in CT^CRISPR^ and NOXA^CRISPR−/−^ MDA-MB-231 in the presence of the pan-caspase inhibitor Q-VD-OPh or not. **F** Imunoblot analysis of caspase-3 expression in CT^CRISPR^ and CASP3^CRISPR−/−^ MDA-MB-231 cells. **G** Kinetics of single cell death analysis, in untreated (UNT) or treated by Taxol (70 nm) or A1331852 (100 nm) alone or combined during 48 h, in CT^CRISPR^ and CASP3^CRISPR−/−^ MDA-MB-231. **H** % of cells showing blebs either associated to secondary vacuole or not, or with a unique vacuole without blebs, after a 48-h exposure to combined treatment in CT^CRISPR^ and CASP3^CRISPR−/−^ MDA-MB-231 cells, including surviving cells (PI negative) in CASP3^CRISPR−/−^ cells at the end of the experiments. **I** Kinetic quantification of Caspase 3/7 activity during treatment by Taxol or A1331852 alone or combined at 18, 24, 36, and 48 h, in CT^CRISPR^ and NOXA^CRISPR−/−^ MDA-MB-231 cells. All experiments were conducted at least 3 times in independent experiments, and results are presented as representative ones with mean values ± SEM.

Since NOXA/MCL-1 complexes modulate MOMP, we evaluated cell death and MOMP onset by quantifying Annexin V/IP positive cells and cyto-c release after 36 h of treatment in NOXA^CRISPR−/−^ cells compared to CT^CRISPR^ cells in both cell lines (Fig. 2A, B and Fig. S1A). We noticed that NOXA gene deletion deeply limited cyto-c release compared to CT^CRISPR^, while cell death rates were comparable. Importantly, a low level of cyto-c release was still detected in NOXA^CRISPR−/−^ cells. The contribution of MOMP to the cell death process was further explored using double KO BAX/BAK MDA-MB-231 cells (Fig. 2C). Videomicroscopy analysis indicated that in these cells, cell death was completely prevented (Fig. 2D), highlighting the major role of MOMP in the cell death process. Pharmacological caspase-3 inhibition using the pan-caspase inhibitor Q-VD-OPh partly decreased cell death rates (Fig. 2E) associated with the apoptotic mode (in control cells) and with the vacuole-associated one (in NOXA^CRISPR−/−^ cells). Interestingly, however, increasing Q-VD-OPh concentrations favored vacuole-associated cell death phenotype at the expense of the apoptotic mode (Fig. S1B). Consistently, deletion of CASP3 (CASP3^CRISPR−/−^cells) (Fig. 2F) protected cells from cell death (Fig. 2G, H) as evidenced by their lack of incorporation of the viability exclusion dye at the end of the experiment. This argues that, while caspase activity contributes to the extent of cell death events in either mode, its intensity determines the type of cell death (with high intensity determining apoptotic mode). In agreement with this, kinetics of caspase-3/7 activities detected during treatments, arose faster in CT^CRISPR^ cells when apoptotic mode prevailed over the vacuole-associated cell one (Fig. 2I). To investigate whether decreasing MOMP intensity would also affect cell death modalities, we decreased the concentration of the BCL-xL targeting BH3 mimetic in combination with Taxol, in the treatment of wild-type cells (Fig. S1C). This promoted vacuole-associated cell death process (from 20% for 100 nM of BH3 mimetic to 55% for 5 nM). Altogether, these results demonstrate that treatment-induced cell death—whether apoptotic or vacuole-associated—requires the onset of MOMP and caspase-3 activity, and that their intensity determines the resulting cell death phenotype.

### GSDME contributes to the vacuole-associated cell death onset in antimitotic-treated cancer cells

**Fig. 3:**
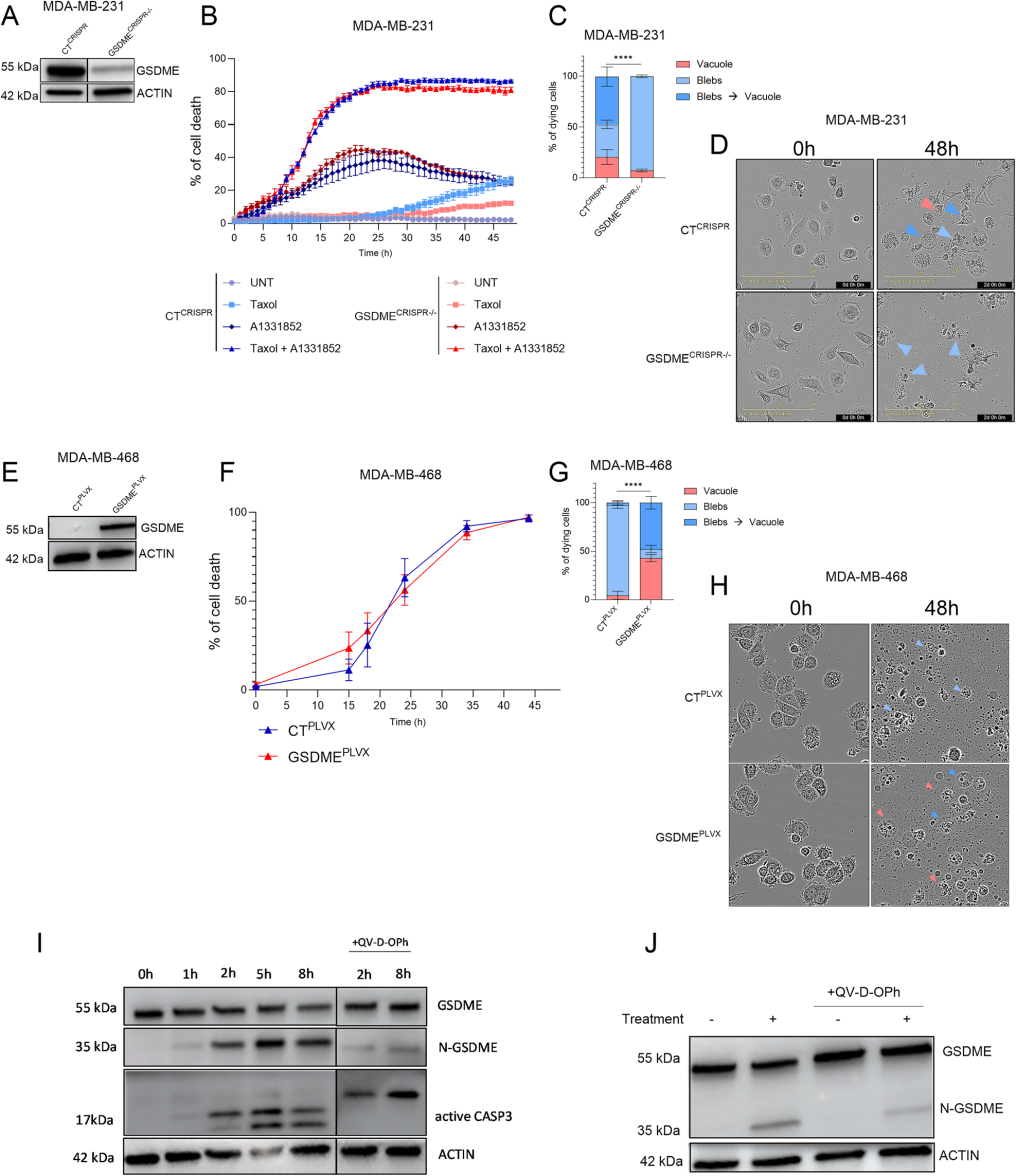
GSDME controls pyroptotic-like cell death induced by antimitotic-based chemotherapy, in correlation with its proteolytic cleavage. **A** Immunoblot analysis of GSDME expression in CT^CRISPR^ and GSDME^CRISPR−/^− MDA-MB-231 cells. **B** Kinetics of single cell death representative analysis in untreated (UNT) or treated by Taxol (70 nm) or the BCL-xL inhibitor A1331852 (100 nm) alone or in combination during 48 h, in CT^CRISPR^ and GSDME^CRISPR−/−^ MDA-MB-231 cells. **C**, **G** % of cells dying with blebs either associated with a secondary vacuole or not, or with a unique vacuole without blebs after a 48-h exposure to combined treatment in CT^CRISPR^ and GSDME^CRISPR−/−^ MDA-MB-231 cells (**C**) and CT^PLVX^ and GSDME^PLVX^ MDA-MB-468 cells (**G**). **D**, **H** Illustrative images at 0 and 48 h of combined treatment with blue arrows pointing to blebs associated with cell death and red arrows pointing to vacuole-associated cell death in CT^CRISPR^ (upper panel) and GSDME^CRISPR−/−^ (lower panel) MDA-MB-231 (**D**) and in CT^PLVX^(upper panel) and GSDME^PLVX^ (lower panel) MDA-MB-468 cells (**H**). **E** Immunoblot analysis of GSDME expression in CT^PLVX^ and GSDME^PLVX^ MDA-MB-468 cells. **F** Kinetics of single cell death in combination of Taxol and BCL-xL inhibitor A1331852 (100 nM) in CT^PLVX^ and GSDME^PLVX^. **I**, **J** Immunoblot analysis of GSDME cleavage upon treatment of Taxol and A1331852 in the presence of pan-caspase inhibitor Q-VD-OPh or not, in MDA-MB-231 (**I**) and GSDME^PLVX^ MDA-MB-468 cells (**J**), respectively. All experiments were conducted at least three times in independent experiments, and results are presented as representative ones with mean values ± SEM.

Given that the vacuole formation observed during cell death may involve a pyroptosis-like execution pathway, we examined members of the gasdermin (GSDM) family, with particular attention to GSDME, which is markedly expressed in MDA-MB-231 cells (Fig. 3A). GSDME gene depletion by CRISPR-Cas9 had no significant impact on cell death kinetics (Fig. 3B), however it mostly prevented the vacuole-associated cell death onset in cancer cells upon cytotoxic treatment, to the benefit of enhanced apoptotic cell death rates (Fig. 3C), as illustrated in Fig. 3D. Of note, deletion of GSDMD in these cells had no significant impact on cell death execution (Fig. S2A–C). To confirm these results, we over-expressed the GSDME protein (GSDME^PLVX^) in the TNBC cell line MDA-MB-468, which does not intrinsically express it (Fig. 3E). Kinetic analysis of cell death showed no difference between both cell lines (Fig. 3F). In contrast, restoring GSDME expression led to vacuole-associated cell death similar to what we observed in the CT^CRISPR^ MDA-MB-231 cells (Fig. 3G), as illustrated in Fig. 3H. Importantly, a cleaved form of GSDME protein, compatible with its N-terminal pore-forming fragment, could be detected by immunoblot in treated cells in both GSDME expressing cell lines, in correlation with active caspase-3 detection (Fig. 3I, J). Moreover, inhibition of apoptotic caspases with Q-VD-OPh partially reduced GSDME cleavage under cytotoxic conditions. These results therefore indicate that GSDME predominantly mediates treatment-induced, vacuole-associated cell death in cancer cells, and that its cleavage depends, at least in part, on concomitant activation of apoptotic caspases.

### Cancer cells preferentially produce immune-regulatory cytokines when the GSDME-dependent cell death program is engaged

**Fig. 4:**
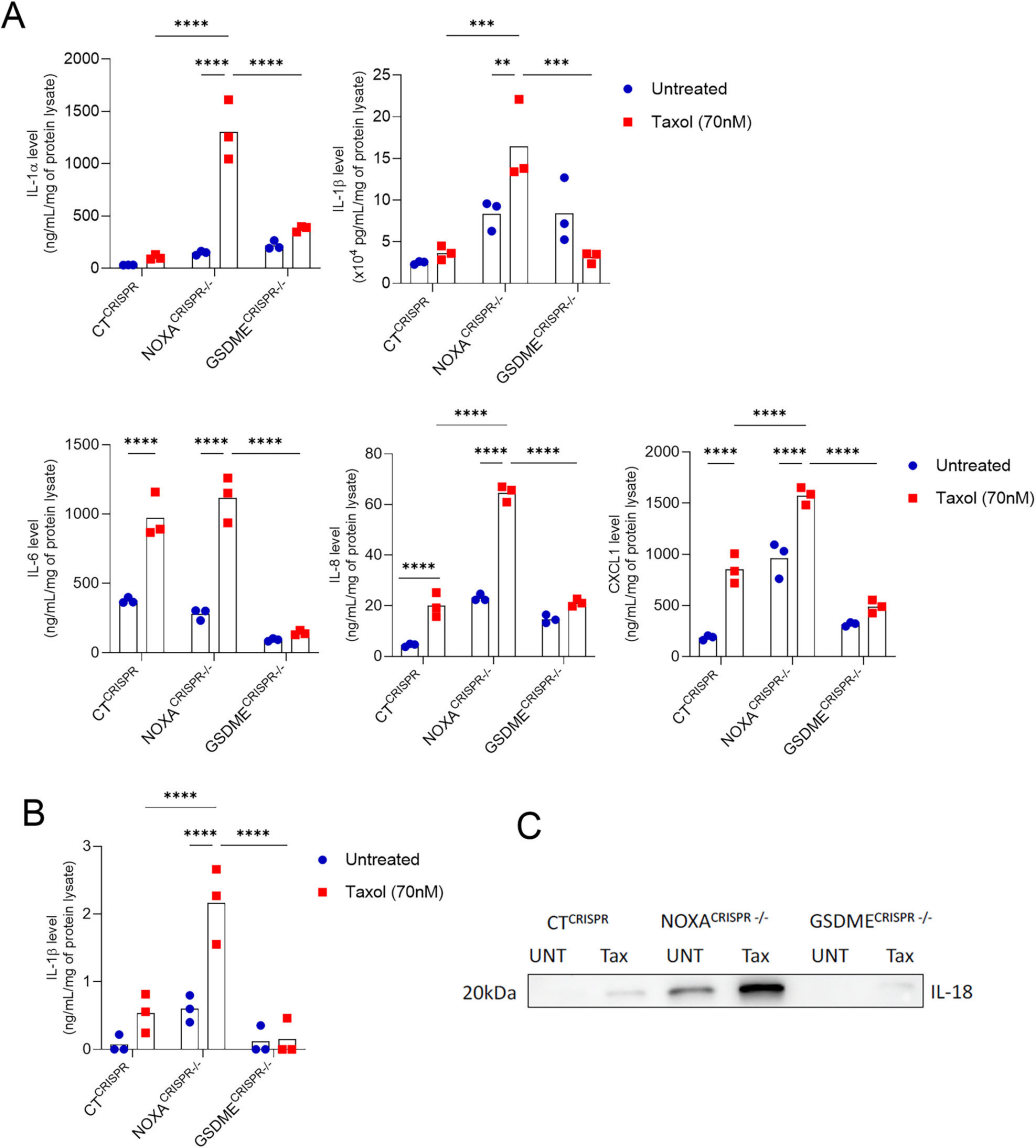
Taxol induced a NOXA and GSDME-dependent pro-inflammatory cytokine secretion. **A** Quantification of IL-1α, IL-1β, IL-6, IL-8, and CXCL1 secretion in 48 h CM from CT^CRISPR^, NOXA^CRISPR-/-^ and GSDME^CRISPR−/−^ MDA-MB-231 cells treated for 24 h or not with Taxol by multiplex cytometry-based technology. **B** IL-1b secretion measured by ELISA in CT^CRISPR^, NOXA^CRISPR−/−^ and GSDME^CRISPR−/−^ MDA-MB-231 cells after Taxol treatment or not. **C** Immunoblot analysis of IL-18 secretion in the supernatants of CT^CRISPR^, NOXA^CRISPR−/−^ and GSDME^CRISPR−/−^ MDA-MB-231 cells treated for 24 h or not with Taxol. All experiments were conducted at least three times in independent experiments, and results are presented as mean values ± SEM when relevant (**A**, **B**).

We then explored more specifically the production of certain cytokines, including those indicative of both the pyroptotic and inflammatory processes. Using a multiplex cytokine assay revealed by spectral flow cytometry, we detected IL-1α and IL-1β secretion in media conditioned by MDA-MB-231 cells when cells are treated with Taxol and NOXA is depleted (Fig. 4A, B). Taxol also induced IL-6, IL-8 and CXCL1 secretion, but both in CT^CRISPR^ and NOXA^CRISPR−/−^cells. Interestingly, IL-1α and IL-1β secretion correlated with cell death modes preferentially triggered by Taxol treatment, with vacuole-associated mode in proficient (NOXA^CRISPR−/−^) cells producing the highest amounts of cytokines after Taxol treatment compared to apoptotic-prone CT^CRISPR^ or GSDME^CRISPR−/−^ cells. We confirmed IL-1β production after Taxol treatment in CM of NOXA^CRISPR−/−^cells by ELISA (Fig. 4B).

We further analyzed secretion of IL-18 in Taxol-treated compared to untreated vacuole-associated cell death or apoptotic-prone cells by immunoblot analysis, revealing higher IL-18 production in CM from Taxol-treated MDA-MB-231 NOXA^CRISPR−/−^ line compared to control or GSDME^CRISPR−/−^ cells (Fig. 4C). We observed that IL-18 could be detected in its short form of 20 kDa corresponding to its mature form resulting from its intracellular proteolytic cleavage. To decipher the mechanism of IL-18 secretion, we investigated the canonical inflammasome pathway involving caspase-1 activity. We thus produced CASP1^CRISPR−/−^ to analyze their ability to secrete IL-18. We could not evidence significant change in either the cell death process (Fig. S3A, B) nor in IL-18 maturation or secretion (Fig. S3C) between CASP1^CRISPR−/−^ and CT^CRISPR^ cells, ruling out a caspase-1 dependent pathway in IL-18 production upon Taxol treatment in these cancer cells. Given that NK cells are responsive to IL-18 [25, 26], we next investigated whether conditioned media (CM) from Taxol-treated cells may modulate their proliferation. To this end, we performed proliferation assays using NK-92 cells exposed to CM derived from Taxol-treated CT or NOXA-depleted cancer cells. We observed that neutralization of IL-18, using either an anti–IL-18 antibody or an IL-18-binding protein, reduced NK cell proliferation in the presence of CM from cells undergoing vacuole-associated cell death (MDA-MB-231 NOXA^CRISPR-/-^), compared with CM from cells undergoing predominantly apoptotic cell death (MDA-MB-231 CT^CRISPR^). In addition, NK cells exposed to Taxol-treated CM from MDA-MB-231 NOXA^CRISPR−/−^, displayed increased ERK1/2 phosphorylation in an IL-18-dependent manner (Supplementary Fig. 4B), consistent with activation of IL-18-dependent signaling pathways.

### Transcription of inflammation-associated *IL1A/B* and *IL18* genes is regulated by antimitotic treatment in breast cancer cells and tumors

**Fig. 5:**
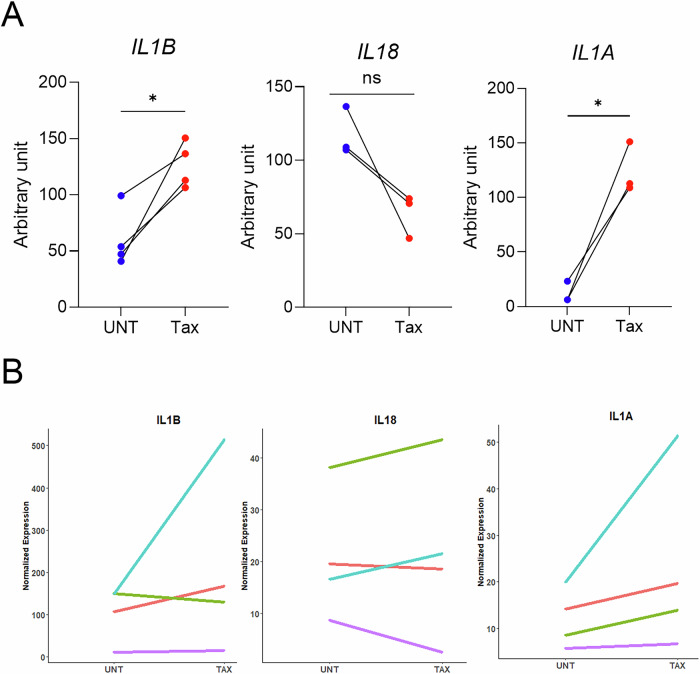
Induction of inflammatory gene transcription by Taxol in established cell lines and primary breast tumors. **A** Q-PCR analysis of IL1A, IL1B, and IL18 mRNA in CT^CRISPR^ MDA-MB-231 cells after Taxol treatment (24 h) or not. **B** Transcriptomic analysis (IL1A, IL1B, and IL18) using the Nanostring Inflammatory genes’ panel after a 48 h ex vivo treatment with Taxol (TAX) or not (UNT) in four human primary breast tumors, among them three Taxol-sensible (red, green, and blue) and one Taxol-resistant (purple). All experiments were conducted at least three times in independent experiments.

Since IL-1α/β and IL-18 secretions were modulated by Taxol treatment and/or the cell death process, we performed qPCR analysis to evaluate the transcriptional activity of their corresponding genes. *IL-1A* and *IL-1B* genes transcription was strongly induced by Taxol in MDA-MB-231 cells (Fig. 5A). In contrast, *IL18* coding mRNAs were not induced in these cells but even repressed by Taxol.

We further interrogated these points in primary tumors, using the Nanostring technology approach. We compared inflammation gene expression in four primary tumors from patients, maintained ex vivo in thin slices for 48 h, with Taxol or not (as described in [18]). The expressions of *IL1B* or *IL1A* genes were increased upon Taxol treatment in three samples out of four tested (Fig. 5B). In contrast, *IL18* gene expression was not or only slightly induced in the Taxol-treated tumors. We could notice that in this panel, the Taxol-resistant tumor (that is the one in which Taxol treatment induced the least active caspase-3 in tumor cells) showed the weakest regulation of *IL1B and IL1A* and a decreased *IL18* transcription upon Taxol treatment.

Altogether, these results indicate that Taxol triggers the production of IL-1α and IL-1β in relation to increased gene transcription, in contrast to IL-18 secretion.

## Discussion

Cell death is a multifaceted process governed by tightly regulated pathways that balance survival and death signals, as well as the inflammatory output of dying cells. Here, we show that antimitotic therapy in breast cancer cells induces a heterogeneous cell death program that can proceed either through classical apoptosis or via a vacuole-associated and still BAX/BAK and Caspase-3 dependent pathway. Our data indicate that the choice between these fates is controlled by the NOXA/MCL-1 axis and GSDME expression and is critical for determining the inflammatory nature of the response. We found that the loss of NOXA not only delays chemotherapy-induced cell death in breast cancer Taxol, but shifts its mode from apoptosis toward a GSDME-dependent pathway associated with inflammatory cytokine release.

The major role of NOXA in the response to anticancer drugs lies in its antagonism of MCL-1 [27]. Our findings reveal an additional and unexpected function of MCL-1 in regulating the crosstalk between chemotherapy-induced cell death pathways, rather than acting solely as a brake on apoptosis initiation. Beyond its established role as a p53-target gene in genotoxic stress [28], NOXA also supports cancer cell death triggered by antimitotics and proteasome inhibitors [18, 29]. Conversely, destabilization of NOXA mRNA by oncogenic driver-targeting therapies such as BRAF or EGFR inhibitors, has been reported as an adaptive mechanism that promotes MCL-1 dependency and therapeutic resistance [30]. Similarly, genomic analysis of residual disease after neoadjuvant chemotherapy in TNBC, have identified *MCL1* gene amplification as one of the most frequent alterations in resistant tumors [31]. Collectively, these data highlight NOXA and MCL-1 as key regulators of anticancer treatment responses.

We further dissected the underlying molecular mechanisms and identified that the temporal coordination of MOMP, cyto-c release, and caspase-3 activation as critical determinants of the executed cell death program. MOMP emerges as a pivotal event, as its genetic ablation through BAX/BAK double KO completely prevented cell death, regardless of whether apoptosis or the vacuole-associated was engaged. In addition, enforced MCL-1 expression, which inhibits MOMP, efficiently suppressed apoptotic cell death, but did not prevent the emergence of vacuole-associated death. Together with the reduced cytochrome-c release and caspase-3 activity observed in NOXA-deficient conditions, these results support the existence of an inflammatory cell death pathway when GSDME is present. This pathway may originate from a slowly progressing apoptotic process that transitions into a secondary necrosis, proposed by Vanden Berghe et al. [24], although it clearly differs from the GSDME-independent secondary necrotic mechanism recently reported by Gavalli et al. [32]. Overall, our results indicate that activation of apoptotic signaling can diverge into multiple execution programs, with distinct inflammatory consequences, underscoring the need for a deeper mechanistic dissection of these processes. Of note, this incomplete execution of apoptosis observed here—insufficient to complete canonical apoptotic dismantling but sufficient to engage GSDME-dependent cell death—suggests a state resembling the “sub-apoptotic MOMP”, as described by Tait and colleagues in which partial MOMP allows survival if caspase activity is blocked [33]. Such limited MOMP may contribute to long-term tumor evolution and resistance, potentially through increased DNA damage driven by Caspase-activated DNAse (CAD) activity and altered mitochondrial dynamics [34, 35]. Conversely, caspase inhibition during MOMP has also been reported to exert anti-tumorigenic effects, partly through NF-kB-dependent regulation of necroptosis and immune responses [36]. Our data support the idea that chemotherapy- induced sub-optimal MOMP may predispose cancer cells to undergo an inflammatory form of cell death. Indeed, mild MOMP was sufficient to drive cells toward the GSDME-dependent vacuole-associated and cytokine-releasing cell death rather than apoptosis. Interestingly, drug-tolerant cancer cell populations arising after sublethal treatments, have also been reported to display increased susceptibility to ferroptosis in some contexts [37], further emphasizing the plasticity of death pathways

We further show that GSDME expression, but not GSDMD, is a decisive determinant of the observed cell death phenotype. Gasdermins are recognized as key executors of pyroptosis, downstream inflammatory caspases [38]. Beyond its proposed tumor suppressive role in breast tumors, through enhancement of antitumor immunity [39], GSDME has been shown to promote pyroptosis in chemotherapy-treated cancer cells, via caspase-3-mediated pore formation [12, 40]. Our data substantiate these findings and highlight caspase-3 as a central upstream regulator of GSDME activation in chemotherapy-induced vacuole-associated cell death. In cells undergoing partial MOMP, GSDME cleavage may thus function as a safeguard against the persistence of genetically unstable cells emerging from CAD activation [41], and its downregulation could facilitate tumor evolution.

Given that distinct cell death modalities have divergent effects on inflammation, we performed multiplex cytokine profiling to compare secretomes from cells undergoing apoptosis versus GSDME-dependent vacuole-associated death, as dictated by NOXA or GSDME status. Cells undergoing the latter cell death mode in response to antimitotic treatment displayed a markedly more pro-inflammatory secretory profile than apoptotic cells, characterized by increased IL-1α, IL-1β, IL-6, IL-8, and CXCL1.

Focusing on IL-1α/β and IL-18, we found that Taxol-induced antimitotic stress promoted their production specifically in the context of GSDME-dependent cell death. This is consistent with prior reports implicating GSDME in IL1β release, particularly in myeloid cells [42, 43]. Regarding IL-18, our data indicate that antimitotic treatment enhances its secretion in breast cancer cells, without a detectable requirement for caspase-1, as its depletion had no measurable effect. This contrasts with previous work reporting caspase-1-dependent IL-18 maturation in murine TNBC cells under chemotherapy stress linked to IRE1a activation [44], suggesting that IL-18 may involve alternative pathways [45]. Notably, *IL18* transcription was not induced by Taxol in either cell lines or in patient-derived tumor samples, despite increased extracellular levels, implying IL-18 release may rely on preexisting intracellular pools rather than de novo synthesis. However, it is important to acknowledge that we were unable to differentiate *IL18* expression by malignant or non-malignant cells within patient tumor slices, and this mechanism requires further investigation. Functionally conditioned media from NOXA-deficient, Taxol-treated cells undergoing GSDME-dependent death, promoted NK cell proliferation in an IL-18-dependent manner. IL-18 is known to modulate immune responses, and in particular, to regulate NK cells' function [46, 47], highlighting its potential clinical relevance and underscoring the need to clarify whether therapeutic targeting of IL-18 would be beneficial or detrimental in cancer treatment.

We also observed increased transcription of *IL1A* and *IL1B* in response after Taxol treatment, in the breast cancer cell line MDA-MB-231 and in three patient-derived tumors exposed to Taxol ex vivo. Although IL-1α and IL-1β can exert both pro- and antitumoral effects depending on context, elevated expression has been associated with improved prognosis in breast cancer, particularly in TNBC. Moreover, preclinical studies suggest that blockade of IL-1 signaling may enhance adaptive antitumor immunity and improve responses to chemotherapy and/or immune checkpoint inhibition [48, 49].

In summary, our data demonstrate that chemotherapy-induced cell death is a major driver of inflammation. Specifically, antimitotic treatment promotes production of IL-1 family cytokines (IL-1α, IL-1β, and IL-18) in a manner dictated by the engaged cell death pathway. These findings support the therapeutic concept of favoring a vacuole-associated, GSDME-dependent inflammatory cell death over apoptosis through modulation of the NOXA/MCL-1 axis, as this may enhance antitumor immune responses.

Given the growing interest in harnessing immunogenic cell death during chemotherapy, rational combinations of chemotherapy and immunotherapy are likely to be particularly effective. The encouraging outcomes observed in breast cancer patients treated with chemotherapy plus immune checkpoint inhibitors in the neoadjuvant setting [50, 51] further underscore the importance of understanding how distinct cell death programs shape inflammatory signaling and antitumor immunity.

### Supplementary information

Dumont et al. original Western blots.

## Supplementary information

Supplementary file

Summary WB

## Data Availability

The data that support the findings of this study are available from the corresponding author, [SB-N], upon reasonable request.

## References

[1] Kroemer G, Galassi C, Zitvogel L, Galluzzi L. Immunogenic cell stress and death. Nat Immunol. 2022;23:487–500. 35145297 10.1038/s41590-022-01132-2

[2] Yatim N, Cullen S, Albert ML. Dying cells actively regulate adaptive immune responses. Nat Rev Immunol. 2017;17:262–75. 28287107 10.1038/nri.2017.9

[3] Yatim N, Jusforgues-Saklani H, Orozco S, Schulz O, Barreira da Silva R, Reis e Sousa C, et al. RIPK1 and NF-κB signaling in dying cells determines cross-priming of CD8⁺ T cells. Science. 2015;350:328–34. 26405229 10.1126/science.aad0395 PMC4651449

[4] Legrand AJ, Konstantinou M, Goode EF, Meier P. The diversification of cell death and immunity: memento mori. Mol Cell. 2019;76:232–42. 31586546 10.1016/j.molcel.2019.09.006

[5] Petroni G, Buqué A, Zitvogel L, Kroemer G, Galluzzi L. Immunomodulation by targeted anticancer agents. Cancer Cell. 2021;39:310–45. 33338426 10.1016/j.ccell.2020.11.009

[6] Bedoui S, Herold MJ, Strasser A. Emerging connectivity of programmed cell death pathways and its physiological implications. Nat Rev Mol Cell Biol. 2020;21:678–95. 32873928 10.1038/s41580-020-0270-8

[7] Barillé-Nion S, Lohard S, Juin PP. Targeting of BCL-2 family members during anticancer treatment: a necessary compromise between individual cell and ecosystemic responses? Biomolecules. 2020;10:1109. 32722518 10.3390/biom10081109 PMC7464802

[8] Juin P, Geneste O, Gautier F, Depil S, Campone M. Decoding and unlocking the BCL-2 dependency of cancer cells. Nat Rev Cancer. 2013;13:455–65. 23783119 10.1038/nrc3538

[9] Vitale I, Pietrocola F, Guilbaud E, Aaronson SA, Abrams JM, Adam D, et al. Apoptotic cell death in disease—current understanding of the NCCD 2023. Cell Death Diff. 2023;30:1097–154. 10.1038/s41418-023-01153-w PMC10130819 37100955

[10] Man SM, Karki R, Kanneganti TD. Molecular mechanisms and functions of pyroptosis, inflammatory caspases and inflammasomes in infectious diseases. Immunol Rev. 2017;277:61–75. 28462526 10.1111/imr.12534 PMC5416822

[11] Shi J, Zhao Y, Wang K, Shi X, Wang Y, Huang H, et al. Cleavage of GSDMD by inflammatory caspases determines pyroptotic cell death. Nature. 2015;526:660–65. 26375003 10.1038/nature15514

[12] Wang Y, Gao W, Shi X, Ding J, Liu W, He H, et al. Chemotherapy drugs induce pyroptosis through caspase-3 cleavage of a gasdermin. Nature. 2017;547:99–103. 28459430 10.1038/nature22393

[13] Newton K, Dixit VM, Kayagaki N. Dying cells fan the flames of inflammation. Science. 2021;374:1076–80. 34822265 10.1126/science.abi5934

[14] Marchi S, Guilbaud E, Tait SWG, Yamazaki T, Galluzzi L. Mitochondrial control of inflammation. Nat Rev Immunol. 2023;23:159–73. 35879417 10.1038/s41577-022-00760-x PMC9310369

[15] Rongvaux A, Jackson R, Harman CC, Li T, West AP, de Zoete MR, et al. Apoptotic caspases prevent the induction of type I interferons by mitochondrial DNA. Cell. 2014;159:1563–77. 25525875 10.1016/j.cell.2014.11.037 PMC4272443

[16] White MJ, McArthur K, Metcalf D, Lane RM, Cambier JC, Herold MJ, et al. Apoptotic caspases suppress mtDNA-induced STING-mediated type I IFN production. Cell. 2014;159:1549–62. 25525874 10.1016/j.cell.2014.11.036 PMC4520319

[17] Zierhut C, Yamaguchi N, Paredes M, Luo JD, Carroll T, Funabiki H. The cytoplasmic DNA sensor cGAS promotes mitotic cell death. Cell. 2019;178:302–315.e23. 31299200 10.1016/j.cell.2019.05.035 PMC6693521

[18] Lohard S, Bourgeois N, Maillet L, Gautier F, Fétiveau A, Lasla H, et al. STING-dependent paracriny shapes apoptotic priming of breast tumors in response to anti-mitotic treatment. Nat Commun. 2020;11:259. 31937780 10.1038/s41467-019-13689-y PMC6959316

[19] Schadt L, Sparano C, Schweiger NA, Silina K, Cecconi V, Lucchiari G, et al. Cancer-cell-intrinsic cGAS expression mediates tumor immunogenicity. Cell Rep. 2019;29:1236–48. 31665636 10.1016/j.celrep.2019.09.065

[20] Bakhoum SF, Ngo B, Laughney AM, Cavallo JA, Murphy CJ, Ly P, et al. Chromosomal instability drives metastasis through a cytosolic DNA response. Nature. 2018;553:467–72. 29342134 10.1038/nature25432 PMC5785464

[21] Li J, Hubisz MJ, Earlie EM, Duran MA, Hong C, Varela AA, et al. Non-cell-autonomous cancer progression from chromosomal instability. Nature. 2023;620:1080–88. 37612508 10.1038/s41586-023-06464-z PMC10468402

[22] An H, Heo JS, Kim P, Lian Z, Lee S, Park J, et al. Tetraarsenic hexoxide enhances the generation of mitochondrial ROS to promote pyroptosis by inducing the activation of caspase-3/GSDME in triple-negative breast cancer cells. Cell Death Dis. 2021;12:159. 33558527 10.1038/s41419-021-03454-9 PMC7870965

[23] Xu T, Wang Z, Liu J, Wang G, Zhou D, Du Y, et al. Cyclin-Dependent Kinase inhibitors function as potential immune regulators via inducing pyroptosis in triple negative breast cancer. Front Oncol. 2022;12:820696. 35756622 10.3389/fonc.2022.820696 PMC9213695

[24] Vanden Berghe T, Vanlangenakker N, Parthoens E, Deckers W, Devos M, Festjens N, et al. Necroptosis, necrosis and secondary necrosis converge on similar cellular disintegration features. Cell Death Differ. 2010;17:922–30. 20010783 10.1038/cdd.2009.184

[25] Senju H, Kumagai A, Nakamura Y, Yamaguchi H, Nakatomi K, Fukami S, et al. Effect of IL-18 on the expansion and phenotype of human natural killer cells: application to cancer immunotherapy. Int J Biol Sci. 2018;14:331–40. 29559850 10.7150/ijbs.22809 PMC5859478

[26] Kalina U, Kauschat D, Koyama N, Nuernberger H, Ballas K, Koschmieder S, et al. IL-18 activates STAT3 in the natural killer cell line 92, augments cytotoxic activity, and mediates IFN-gamma production by the stress kinase p38 and by the extracellular regulated kinases p44erk-1 and p42erk-21. J Immunol. 2000;165:1307–13. 10903731 10.4049/jimmunol.165.3.1307

[27] Dumont A, Lohard S, Maillet L, Juin PP, Barillé-Nion S. Noxa the BCL-2 family member behind the scenes in cancer treatment. J Cell Signal. 2020;1:127–43.

[28] Oda E, Ohki R, Murasawa H, Nemoto J, Shibue T, Yamashita T, et al. Noxa, a BH3-only member of the Bcl-2 family and candidate mediator of p53-induced apoptosis. Science. 2000;288:1053–8. 10807576 10.1126/science.288.5468.1053

[29] Qin JZ, Ziffra J, Stennett L, Bodner B, Bonish BK, Chaturvedi V, et al. Proteasome inhibitors trigger NOXA-mediated apoptosis in melanoma and myeloma cells. Cancer Res. 2005;65:6282–93. 16024630 10.1158/0008-5472.CAN-05-0676

[30] Montero J, Gstalder C, Kim DJ, Sadowicz D, Miles W, Manos M, et al. Destabilization of NOXA mRNA as a common resistance mechanism to targeted therapies. Nat Commun. 2019;10:5157. 31727958 10.1038/s41467-019-12477-y PMC6856172

[31] Balko JM, Giltnane JM, Wang K, Schwarz LJ, Young CD, Cook RS, et al. Molecular profiling of the residual disease of triple-negative breast cancers after neoadjuvant chemotherapy identifies actionable therapeutic targets. Cancer Discov. 2014;4:232–45. 24356096 10.1158/2159-8290.CD-13-0286 PMC3946308

[32] Gavali S, Maremonti F, Tonnus W, Gembardt F, Nastassja Schlecht M, Flade K, et al. Secondary necrosis following caspase-activation can occur independently of gasdermin E. Adv Sci. 2025;e07381. 10.1002/advs.202507381 PMC12697764 41185624

[33] Tait SW, Parsons MJ, Llambi F, Bouchier-Hayes L, Connell S, Muñoz-Pinedo C, et al. Resistance to caspase-independent cell death requires persistence of intact mitochondria. Dev Cell. 2010;18:802–13. 20493813 10.1016/j.devcel.2010.03.014 PMC3004027

[34] Ichim G, Lopez J, Ahmed SU, Muthalagu N, Giampazolias E, Delgado ME, et al. Limited mitochondrial permeabilization causes DNA damage and genomic instability in the absence of cell death. Mol Cell. 2015;57:860–72. 25702873 10.1016/j.molcel.2015.01.018 PMC4352766

[35] Cao K, Riley JS, Heilig R, Montes-Gómez AE, Vringer E, Berthenet K, et al. Mitochondrial dynamics regulate genome stability via control of caspase-dependent DNA damage. Dev Cell. 2022;57:1211–25. 35447090 10.1016/j.devcel.2022.03.019 PMC9616799

[36] Giampazolias E, Zunino B, Dhayade S, Bock F, Cloix C, Cao K, et al. Mitochondrial permeabilization engages NF-κB-dependent anti-tumour activity under caspase deficiency. Nat Cell Biol. 2017;19:1116–29. 28846096 10.1038/ncb3596 PMC5624512

[37] Kalkavan H, Chen MJ, Crawford JC, Quarato G, Fitzgerald P, Tait SWG, et al. Sublethal cytochrome c release generates drug-tolerant persister cells. Cell. 2022;185:3356–74. 36055199 10.1016/j.cell.2022.07.025 PMC9450215

[38] Shi J, Gao W, Shao F. Pyroptosis: Gasdermin-mediated programmed necrotic cell death. Trends Biochem Sci. 2017;42:245–54. 27932073 10.1016/j.tibs.2016.10.004

[39] Zhang Z, Zhang Y, Xia S, Kong Q, Li S, Liu X, et al. Gasdermin E suppresses tumour growth by activating anti-tumour immunity. Nature. 2020;579:415–20. 32188940 10.1038/s41586-020-2071-9 PMC7123794

[40] Rogers C, Erkes DA, Nardone A, Aplin AE, Fernandes-Alnemri T, Alnemri ES. Gasdermin pores permeabilize mitochondria to augment caspase-3 activation during apoptosis and inflammasome activation. Nat Commun. 2019;10:1689. 30976076 10.1038/s41467-019-09397-2 PMC6459836

[41] Haimovici A, Höfer C, Badr MT, Bavafaye Haghighi E, Amer T, Boerries M, et al. Spontaneous activity of the mitochondrial apoptosis pathway drives chromosomal defects, the appearance of micronuclei and cancer metastasis through the Caspase-activated DNAse. Cell Death Dis. 2022;13:315. 35393399 10.1038/s41419-022-04768-y PMC8990075

[42] Zhou B, Abbott DW. Gasdermin E permits interleukin-1 beta release in distinct sublytic and pyroptotic phases. Cell Rep. 2021;35:108998. 33852854 10.1016/j.celrep.2021.108998 PMC8106763

[43] Afonina IS, Müller C, Martin SJ, Beyaert R. Proteolytic processing of interleukin-1 family cytokines: variations on a common theme. Immunity. 2015;42:991–1004. 26084020 10.1016/j.immuni.2015.06.003

[44] Xu L, Peng F, Luo Q, Ding Y, Yuan F, Zheng L, et al. IRE1α silences dsRNA to prevent taxane-induced pyroptosis in triple-negative breast cancer. Cell. 2024;187:7248–66. 39419025 10.1016/j.cell.2024.09.032 PMC11645245

[45] Rébé C, Ghiringhelli F. Interleukin-1β and cancer. Cancers. 2020;12:1791. 32635472 10.3390/cancers12071791 PMC7408158

[46] Molgora M, Supino D, Mavilio D, Santoni A, Moretta L, Mantovani A, et al. The yin-yang of the interaction between myelomonocytic cells and NK cells. Scand J Immunol. 2018;88:e12705. 30048003 10.1111/sji.12705 PMC6485394

[47] Park IH, Yang HN, Lee KJ, Kim TS, Lee ES, Jung SY, et al. Tumor-derived IL-18 induces PD-1 expression on immunosuppressive NK cells in triple-negative breast cancer. Oncotarget. 2017;8:32722–30. 28415798 10.18632/oncotarget.16281 PMC5464822

[48] Kaplanov I, Carmi Y, Kornetsky R, Shemesh A, Shurin GV, Shurin MR, et al. Blocking IL-1β reverses the immunosuppression in mouse breast cancer and synergizes with anti-PD-1 for tumor abrogation. Proc Natl Acad Sci USA. 2019;116:1361–69. 30545915 10.1073/pnas.1812266115 PMC6347724

[49] Hänggi K, Li J, Gangadharan A, Liu X, Celias DP, Osunmakinde O, et al. Interleukin-1α release during necrotic-like cell death generates myeloid-driven immunosuppression that restricts anti-tumor immunity. Cancer Cell. 2024;42:2015–31. 39577420 10.1016/j.ccell.2024.10.014 PMC11631672

[50] Bardia A, Hurvitz SA, Tolaney SM, Loirat D, Punie K, Oliveira M, et al. Sacituzumab Govitecan in metastatic triple-negative breast cancer. N Engl J Med. 2021;384:1529–41. 33882206 10.1056/NEJMoa2028485

[51] Shahbandi A, Chiu FY, Ungerleider NA, Kvadas R, Mheidly Z, Sun MJS, et al. Breast cancer cells survive chemotherapy by activating targetable immune-modulatory programs characterized by PD-L1 or CD80. Nat Cancer. 2022;3:1513–33. 36482233 10.1038/s43018-022-00466-y PMC9923777

