# Supplementary material for: NOXA/MCL-1 axis determines cell-death decision between apoptosis and a GSDME-dependent cell death associated with production of inflammatory cytokines upon treatment of breast cancer cells with antimitotics
Source: Cell Death Dis. 2026 Jun 11;17(1):740. doi: 10.1038/s41419-026-08965-x (PMC13482291; doi:10.1038/s41419-026-08965-x)
Supplement: Supplementary file 1 [file 41419_2026_8965_MOESM2_ESM.pdf]

Figure1

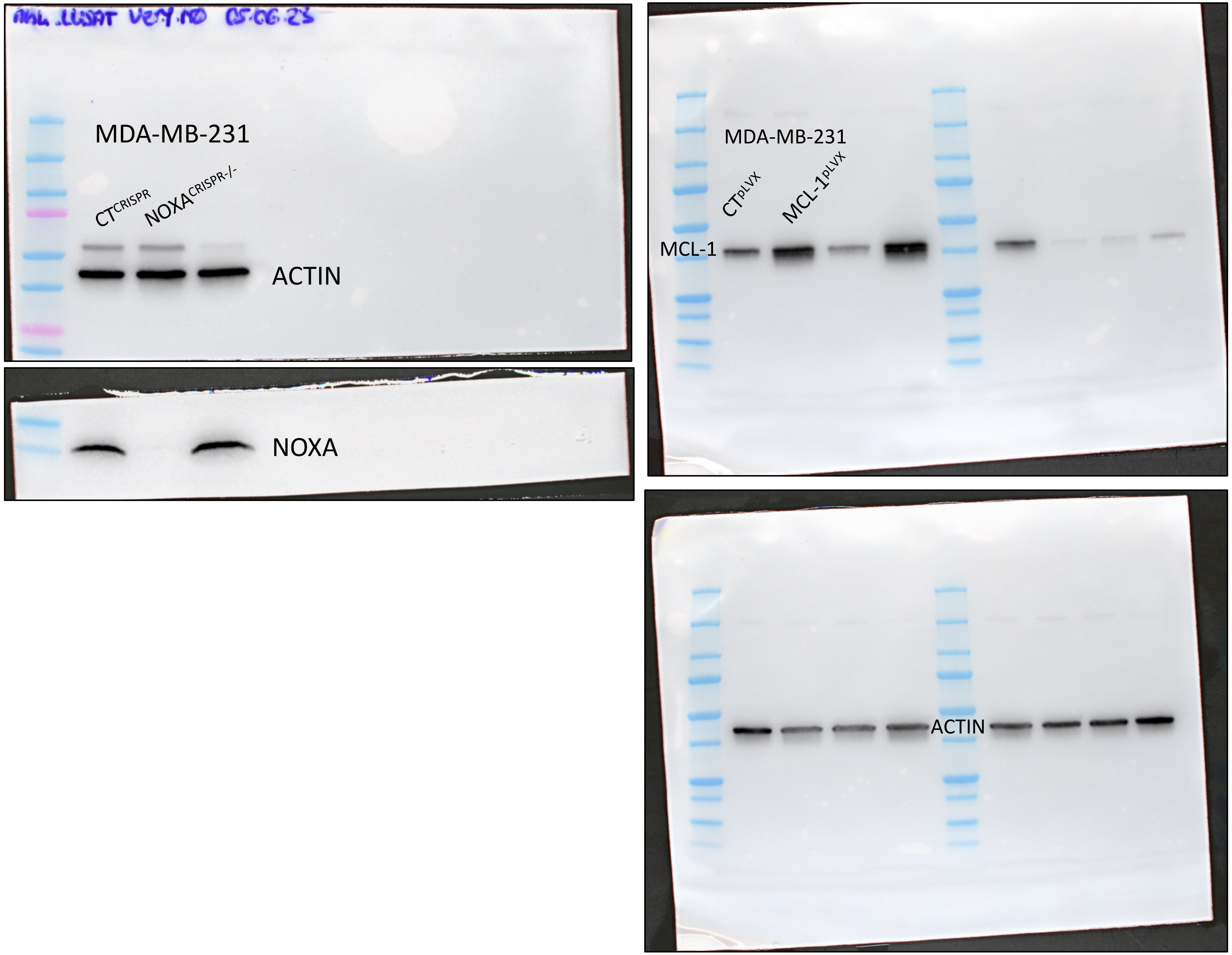
MDA-MB-231
CT CRISPR
NOXA CRISPR-/-
ACTIN
NOXA
MDA-MB-231
CT pLVX
MCL-1 pLVX
MCL-1
ACTIN

Figure2

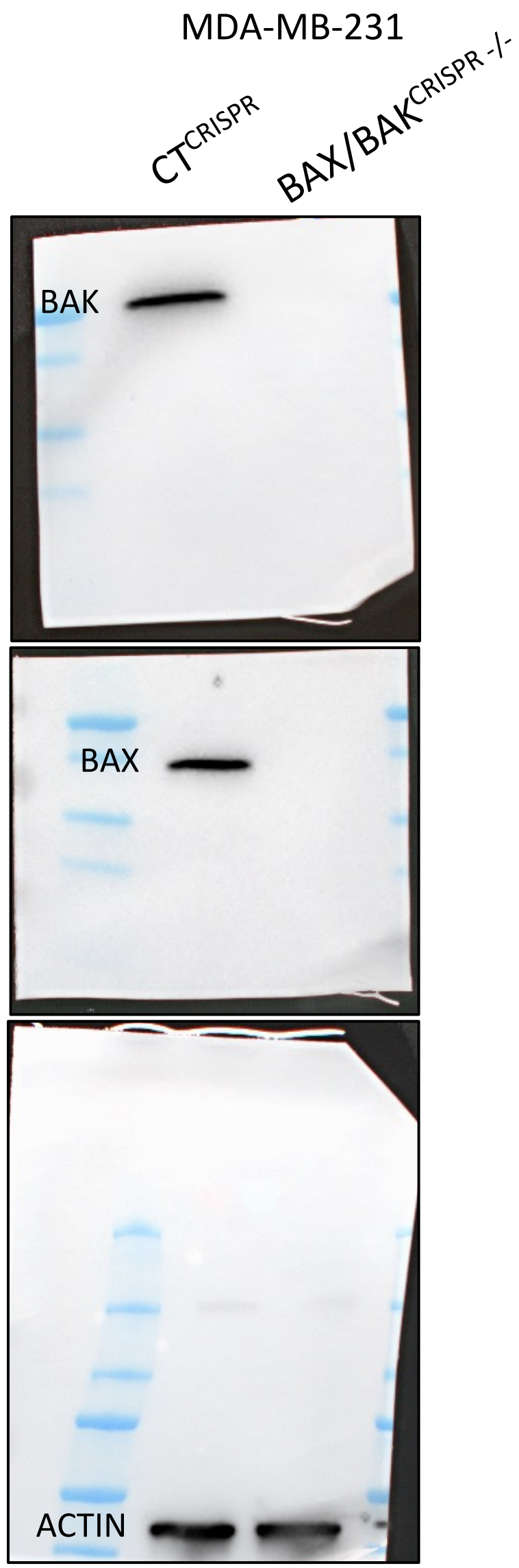
MDA-MB-231
CT^{CRISPR}
BAX/BAK$^{CRISPR -/-}$
BAK
BAX
ACTIN

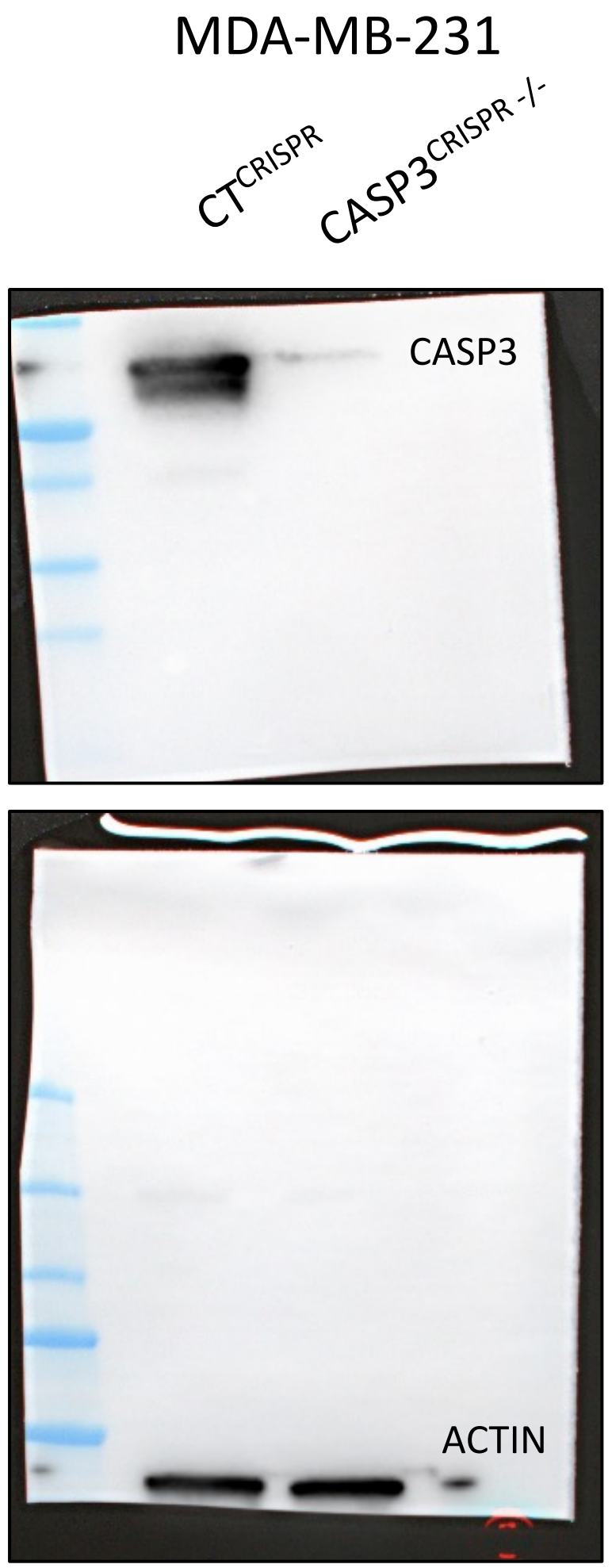
MDA-MB-231
CT^{CRISPR}
CASP3$^{CRISPR -/-}$
CASP3
ACTIN

Figure3

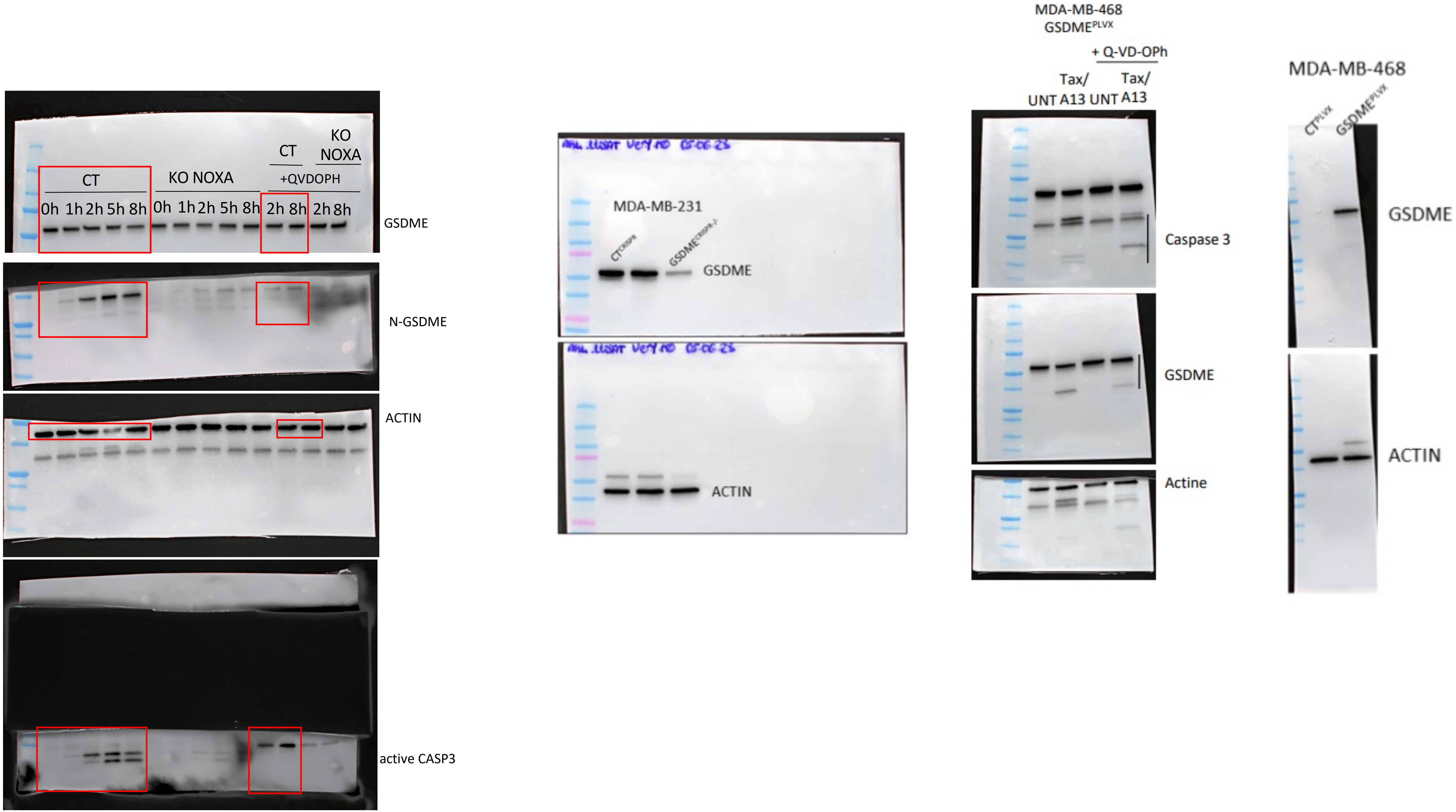

CT
KO NOXA
CT
KO NOXA
+QVDOPH
0h 1h 2h 5h 8h
0h 1h 2h 5h 8h
2h 8h
2h 8h
GSDME
N-GSDME
ACTIN
active CASP3
MDA-MB-231
CTCRISPR
GSDMECRISPR-/-
GSDME
ACTIN
MDA-MB-468
GSDMEPLVX
+ Q-VD-OPh
UNT
Tax/A13
UNT
Tax/A13
Caspase 3
GSDME
Actine
MDA-MB-468
CTPLVX
GSDMEPLVX
GSDME
ACTIN

Figure4

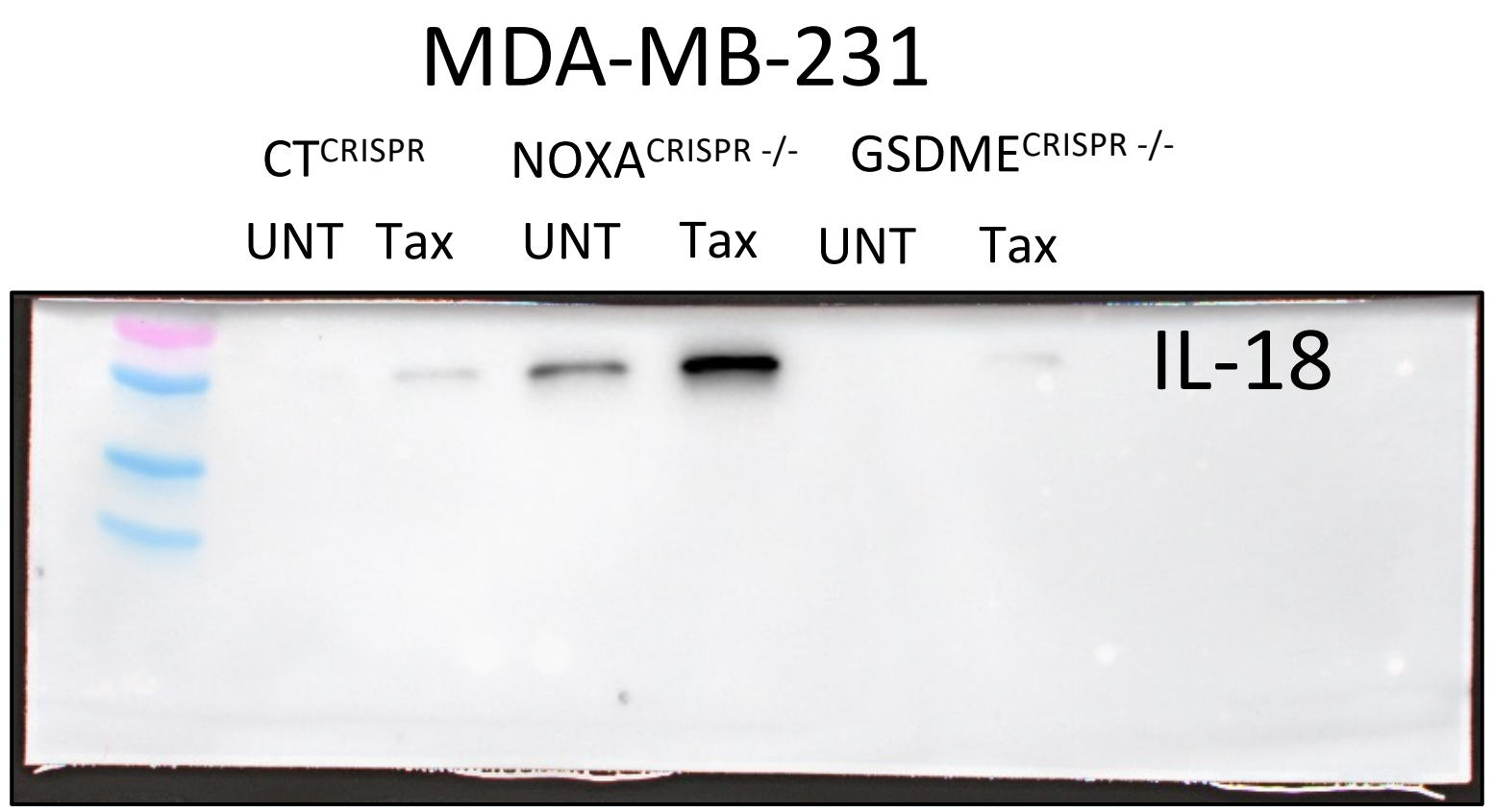
MDA-MB-231
CT^CRISPR
NOXA^CRISPR -/-
GSDME^CRISPR -/-
UNT Tax UNT Tax UNT Tax
IL-18

Supp2

55 kDa

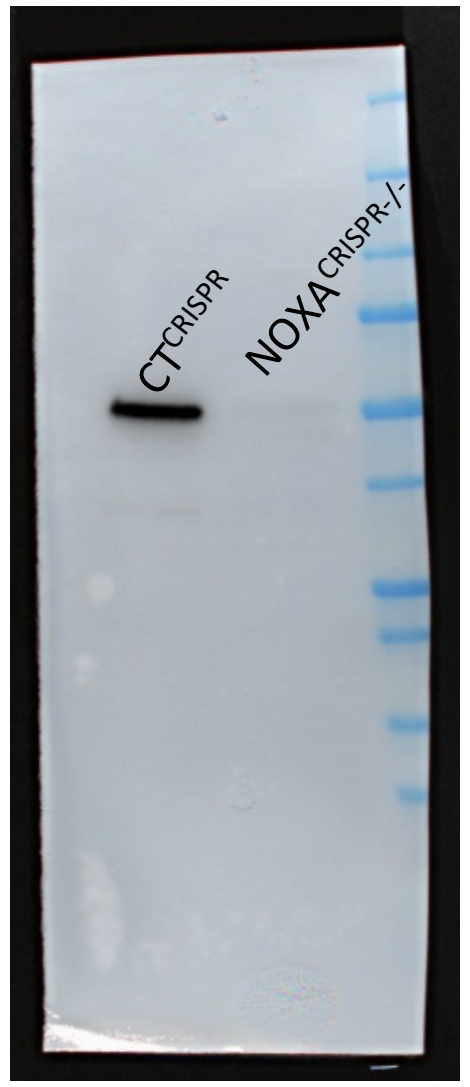

GSDMD

42 kDa

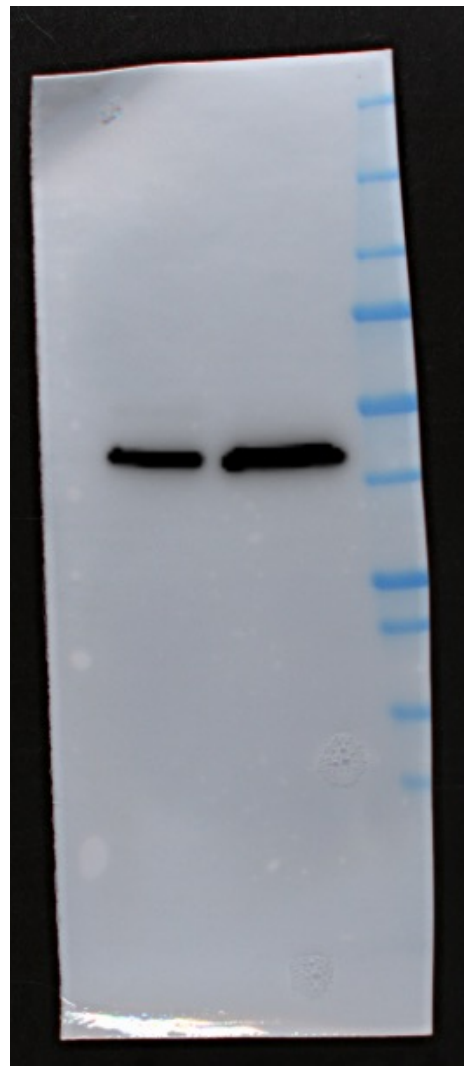

ACTIN

Supp3

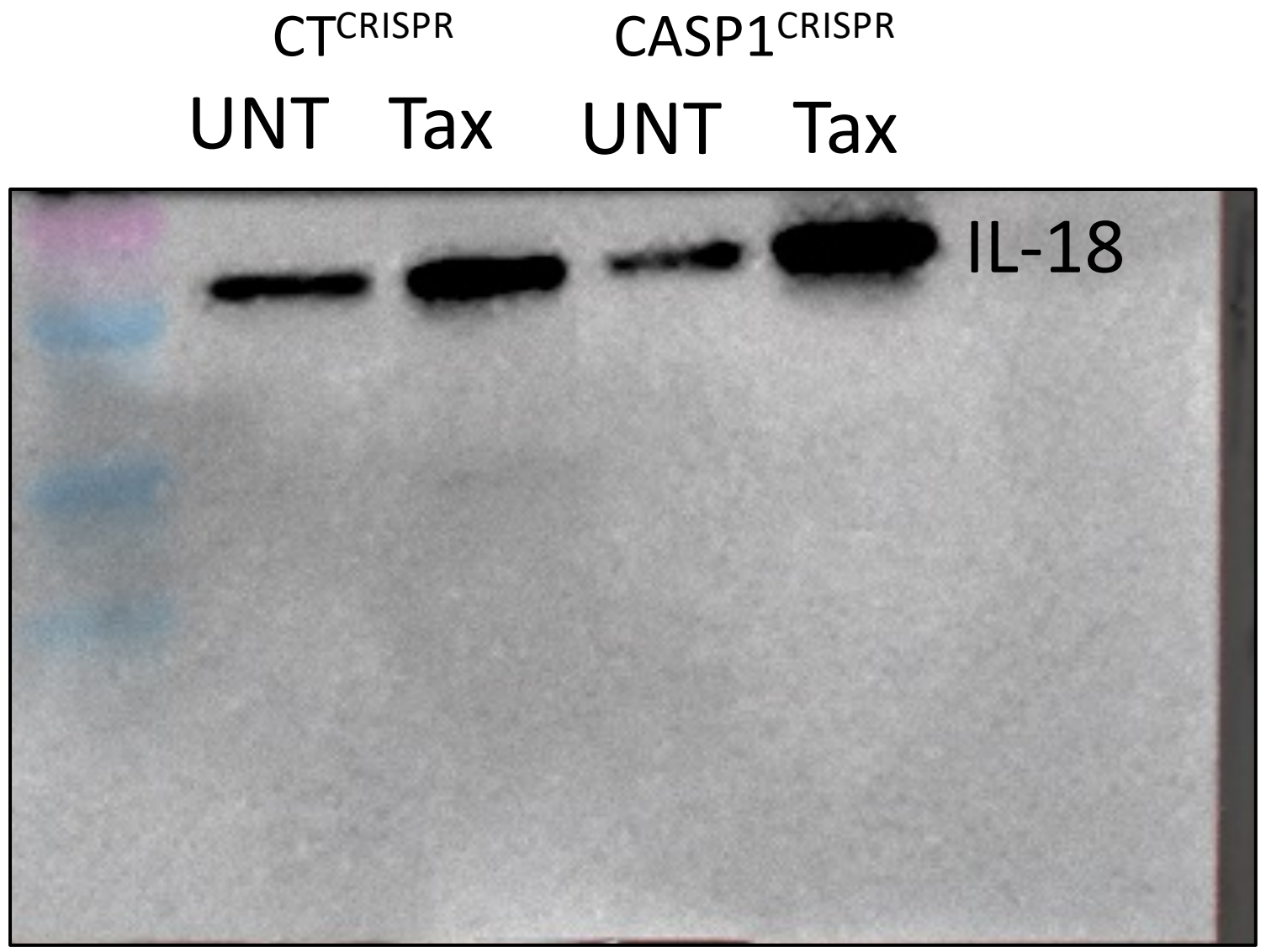

Supp4

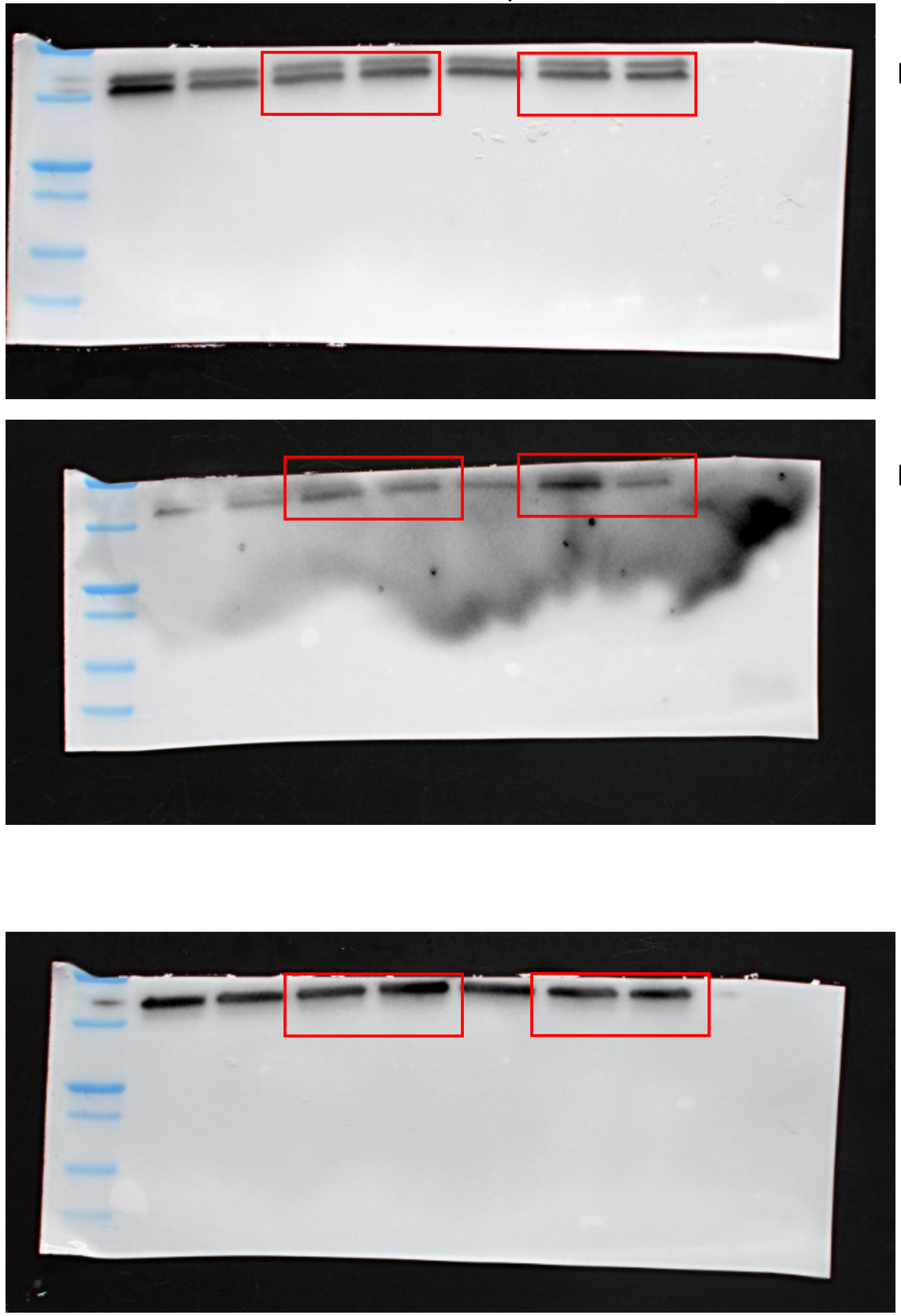
SVF 0%
CTL NT
CTL TAX
CTL TAX + Anti IL-18
Ko NOXA NT
Ko NOXA Tax
Ko NOXA Tax + Anti IL18
42/44 kDa
ERK1/2
42/44 kDa
P-ERK1/2
42 kDa
ACTIN
