# Supplementary material for: NOXA/MCL-1 axis determines cell-death decision between apoptosis and a GSDME-dependent cell death associated with production of inflammatory cytokines upon treatment of breast cancer cells with antimitotics
Source: Cell Death Dis. 2026 Jun 11;17(1):740. doi: 10.1038/s41419-026-08965-x (PMC13482291; doi:10.1038/s41419-026-08965-x)
Supplement: Supplementary file 2 [file 41419_2026_8965_MOESM1_ESM.docx]

**SUPPLEMENTARY MATERIALS and METHODS**

**Proliferation of NK cells**

NK92 cell line was obtained from Enora Ferron (Research Laboratory Etablissement Français du Sang, 44011, Nantes, France) and maintained in RPMI-1640 supplemented with 2 mM glutamine, 1% penicillin/streptomycin, rhIL2 (200UI/ml) and 10% FBS. For proliferation assays, 30 000 NK92 cells were incubated with conditioned media (CM) from 48h Taxol-treated MDA-MB-231 CT^CRISPR^ or NOXA^CRISPR-/-^ cells, in presence or not of blocking IL-18 (either using IL-18Rβ neutralizing antibody (MAB1181, 1μg/ml) or rhIL-18BPa antagonist protein (119-BP, 100ng/ml) from R&D Systems (Bio-Techne, France). Viable cells were counted after 8 days of culture and % of residual proliferation in presence of IL-18 blocking compared to corresponding control condition, was calculated.

Immunoblot analysis of ERK pathway was also performed in these cells upon the same conditions.

**LEGENDS TO SUPPLEMENTARY FIGURES**

**Supplementary Fig 1**

**A** After a 36h-exposure to Taxol + A1331852 treatment, CT^CRISPR^ and NOXA^CRISPR-/-^cells were analyzed for cyto-c expression by flow cytometry to detect cyto-c release. Fluorescence profiles from 3 independent experiments are shown.

**B** Morphological cell death upon increasing concentrations of Q-VD-OPh, was analyzed (as previously shown) in MDA-MB-231 cells using Incucyte imaging.

**C** Morphological cell death upon decreasing concentrations of A1331852, in MDA-MB-231 cells using Incucyte imaging

**Supplementary Fig 2	GSDMD KO did not affect cell death process and cytokine secretion upon Taxol treatment**

**A** Kinetics of single cell death representative analysis in not treated (UNT) or treated by Taxol + A1331852 in combination during 48h, in CT^CRISPR^ and GSDMD^CRISPR-/-^ MDA-MB-231 cells.

**B** % of cells dying with blebs either associated to secondary vacuole or not, or with a unique vacuole without blebs after a 48h-exposure to combined treatment in CT^CRISPR^ and GSDMD^CRISPR-/-^ MDA-MB-231.

All experiments were conducted at least 3 times in independent experiments and results are presented as representative ones with mean values ± SEM.

**Supplementary Fig 3	CASP1 KO did not affect cell death process and cytokine secretion upon Taxol treatment**

**A** Kinetics of single cell death representative analysis in not treated (UNT) or treated by Taxol + A1331852 in combination during 48h, in CT^CRISPR^ and CASP1^CRISPR-/-^ MDA-MB-231 cells.

**B** % of cells dying with blebs either associated to secondary vacuole or not, or with a unique vacuole without blebs after a 48h-exposure to combined treatment in CT^CRISPR^ and CASP1^CRISPR-/-^ MDA-MB-231.

**C** Immunoblot analysis of IL-18 secretion in CT^CRISPR^ and CASP1^CRISPR-/-^ MDA-MB-231.

All experiments were conducted at least 3 times in independent experiments and results are presented as representative ones with mean values ± SEM.

**Supplementary Fig 4	Taxol-induced IL-18 secretion contributes to NK cells proliferation**

**A** IL-18-blocking reduced NK92 cells proliferation (assessed by cell counting) when exposed to Taxol-treated CM from MDA-MB-231 NOXA^CRISPR-/-^ cells but not from CT^CRISPR^ cells.

**B** Immunoblot analysis of ERK pathway indicated its activation in NK92 cells after a 48h exposure to Taxol-treated CM from MDA-MB-231 CT^CRISPR^ and NOXA^CRISPR-/-^ cells.

**SUPPLEMENTARY FIGURES**
